# Naturalistic Decision-Making in Sport: How Current Advances Into Recognition Primed Decision Model Offer Insights for Future Research in Sport Settings?

**DOI:** 10.3389/fpsyg.2022.936140

**Published:** 2022-06-20

**Authors:** Cyril Bossard, Thibault Kérivel, Sylvain Dugény, Pierre Bagot, Tanguy Fontaine, Gilles Kermarrec

**Affiliations:** Research Center for Education Learning and Didactics (EA 3875), University of West Brittany, Brest, France

**Keywords:** decision-making, intuition, recognition, expertise, sports

## Introduction

The Naturalistic Decision-Making (NDM) approach (Klein, 2008, 2015; Hallé Petiot et al., 2021) is an alternative to the cognitivist approach (i.e., decision-making viewed as a rationalistic linear information process) and to the ecological-dynamic approach (i.e., decision-making viewed as behavioral adaptation to environmental constraints). NDM researchers have discovered that decision makers in natural settings rely heavily on intuition. Experts use their experiences to focus their perceptions onto salient features, to recognize situations as typical, and to choose the most appropriate option. The Recognition Primed-Decision (RPD) model (Klein et al., 2010) postulates that experts' decision-making is a recognition process and suggests three levels of experiencing a situation: (i) simple match (recognize a situation and associate the first adequate option), (ii) diagnose the situation (faced with a non-familiar situation and need time to adapt a typical action), (iii) evaluate a course of action (simulate the relevance of the first option through mental visualization).

The RPD model has been applied in sport settings as rapid decision-making is essential for sporting expertise (for a review see, Macquet, 2020). Numerous qualitative studies have investigated the recognition processes in Australian Football officials (Neville et al., 2017), soccer referees (Mascarenhas et al., 2009), badminton players (Macquet and Fleurance, 2007), volleyball players (Macquet, 2009; Fortin-Guichard et al., 2021), soccer players (Bossard, 2010; Kermarrec and Bossard, 2014), ice hockey players (Bossard et al., 2010; Mulligan et al., 2012), handball goalkeepers (Le Menn et al., 2019), basket-ball players (Macquet and Kragba, 2015), judo athletes (Macquet and Lacouchie, 2017), karate athlete (Milazzo and Fournier, 2015), rugby coaches (Collins et al., 2016; Ashford et al., 2020), and sport adventure coaches (Collins and Collins, 2016).

Thus, this consistent empirical evidence obtained through investigation during naturalistic games strengthen the assumption that experts' decision-making in sport is based on their intuition. Nevertheless, some paradoxes remain and call for future investigation: six key points could be promising avenues of research.

## Interplay Between Intuitive and Analytic Decisions

According to Macquet (2020) literature review of the RPD model in sport, 60–81% of the experts' decisions were classified as intuitive, using simple matching, about 13–28% of the experts' decisions were related to diagnosis, and about 3–24% were related to evaluating a course of action. Time pressure in the game situation is invoked to explain the predominance of intuition. Nevertheless, some issues suggest that the role of the decision-maker and the context in which he is embedded could affect the recognition mechanism. Investigating a handball goalkeeper's decision-making (Le Menn et al., 2019) or soccer defenders' decision-making (Kermarrec and Bossard, 2014), researchers found that when athletes were far from the ball, they had more time for mental simulation and diagnosing the development of possible situations. Furthermore, rugby coaches' decision-making was less subject to less time pressure than athletes, so that deliberative or analytic decision were frequent (Collins et al., 2016).

Taken together, these outcomes suggest that decision-making in a sport setting may take place on a continuum between intuitive and analytical processes (Kahneman and Klein, 2009). In this sense, integrated theoretical models of decision-making have recently been proposed for athletes (Ashford et al., 2021a,b; Hallé Petiot et al., 2021), referees (Kittel et al., 2021a; Samuel et al., 2021) and coaches (Collins et al., 2016; Richards et al., 2017). There is a lack of consistent empirical evidence in a sport setting for establishing the continuum from intuitive to analytical decision-making. Furthermore, based on outcomes in other research fields (e.g., Calabretta et al., 2017), future studies in sport should examine the interplay between intuitive and analytic decision-making in athletes, coaches and referees.

## Visual Search Strategies and Intuition

Moreover, NDM studies also brought information on the relevant cues decision-makers take into account when embedded in the course of an action. As pointed by Macquet (2020), relevant cues were context-specific and were mostly concerned with visual perception. Today the field of visual search strategies is fertile among athletes (Vater et al., 2019; Krabben et al., 2022), referees (Luis del Campo et al., 2018; Moore et al., 2019) or coaches (Robertson et al., 2018; Mitchell et al., 2020). New methodological designs need to be proposed in order to study the visual information that is prioritized by experts in situation recognition and intuitive decisions in more ecological conditions (i.e., naturalistic conditions). Research designs that immerse participants in environments close to real sports settings with video filmed with a 360° camera (Musculus et al., 2021) or with virtual reality (Mascret et al., 2022) seem to be a very promising methodological avenue. Thus, an original contribution from RPD for sports may consist of studying decision-making in naturalistic sports games settings, providing empirical evidence on the way experts make intuitive decision and use very few cues to support ongoing decisions and actions.

## Intuitive Decision-Making and Creativity

Although it is common to associate intuition and creativity, a clear link between them has not been adequately established in sports sciences. Quite the contrary: previous empirical research on IDM in sports settings showed that expert athletes choose their preferred “first option” most of time (in 80% of the decisions made), so they could be considered as non-creative. Nevertheless, the role of intuition in creativity should not be neglected as it is often reported to be a core component of the idea generation process (Pétervári et al., 2016). Sports researchers recently took into consideration this assumption when experimenting team sports programs aiming to develop players' creativity and adaptive behaviors (e.g., Coutinho et al., 2016). Furthermore, the relation between creativity and perceptual and cognitive processes was recently examined in experimental decision-making studies. Firstly, creative decision-making and visual search behavior were investigated in skilled soccer players (Roca et al., 2018; Roca and Ford, 2020). The results showed that most-creative players employed a broader attentional focus including more fixations of shorter duration and toward more informative locations of the display compared with least-creative players. Secondly, Klatt et al. (2019) examined the relation between creativity and IDM in two studies—one involving coaches and one involving soccer players—using video footage of real soccer matches. Results indicate a positive correlation between a player's creativity score and the quality of the first generated option for the whole sample. Even if the topics of those studies were not the recognition primed-decision processes, the results confirm insights about the relationship between IDM and creativity. If more creative players and coaches tend to maintain a broader attentional focus in a realistic sport setting, they should also be more sensitive to recognizing many salient cues.

## IDM and the Role of Emotions

In recent decades, interest in the links between decision-making processes and emotions has grown (George and Dane, 2016). Emotions have often been considered as biases or hindrances to decision-making, especially in cognitive approaches studying athletes, referees and coaches (Tenenbaum et al., 2013). More recently, emotions have begun to emerge as resources for decision-making in a variety of models (e.g., Affect as information approaches, Appraisal tendency framework, Affect infusion model). The NDM approach also considers emotion as a potential resource. Empirical studies on this issue have focused on managers in companies (Sayegh et al., 2004), bank agents (Lipshitz and Shulimovitz, 2007), emergency doctors (Coget and Keller, 2010), film directors (Coget et al., 2011) or aircraft maintenance engineers (Naweed and Kingshott, 2019). These studies highlight the diverse roles of emotions in intuitive processes involved in complex, uncertain and time-pressured situations, supporting the theoretical assumptions of the NDM approach (Mosier and Fischer, 2010). To our knowledge, no study has shown the role of emotions in IDM in the context of sport. According to several empirical studies cited above and conducted through the NDM framework, situationally-induced emotions in a real-world context, namely “integral affects” (Mosier and Fischer, 2010) could play different roles in sports decision-making. Taken together these issues suggest further investigation of the role of emotion in the RPD model in a naturalistic decision-making perspective. Three hypotheses concerning these roles can thus be proposed: (1) expert pattern matching and intuitive decisions often could be guided by an affective appraisal of the situation, (2) emotional responses such as discomfort could motivate individuals to look for more sources of information to make a more deliberative decision, and (3) if the mental simulation of the potential evolution of the situation induces discomfort, individuals might be tempted to opt for another option.

## The Development of IDM

The development of sports experts' IDM underlies two types of research questions corresponding to two distinct promising research avenues: (1) How does the development of IDM contribute to the development of sports expertise? (2) Which training programs could enhance IDM in sport?

NDM researchers in sport science have typically focused on IDM in experts, but the development of intuition and its contribution to the development of expertise still lacks empirical evidence (Martindale and Collins, 2013). Researchers from other fields have provided insights by considering the interaction between intuition and deliberation and how their use might lead to “skilled intuition” (Kahneman and Klein, 2009). Baylor (2001) argued for the possibility that the development of intuition follows a U-shaped progression where immature intuitions start at a relatively high level and then decrease when decisions become more analytic, and later increases with available intuitions. Bangert et al. (2014) hypothesized that musicians deliberately explore and test interpretative options, perhaps guided in their search by intuitive pattern recognition. Future studies in a sports context should examine the development of intuition to understand how athletes, coaches and referees become experts.

Proposals for training decision-making in sport are mainly based on a perceptual-cognitive approach to decision-making both for athletes (Hadlow et al., 2018), referees (Kittel et al., 2021b) and coaches (Richards et al., 2017). Proposals for training IDM based on NDM approach are less common and still lack empirical evidence. The research work of Kittel et al. (2021b) and the recommendations for introducing reflective practices with referees could be an interesting way to improve IDM. Future interventional studies could be set up based on programs dedicated to improving IDM in order to test their effects.

## IDM in Teams

Previous sections in this paper argued to revisit the RPD model (Macquet, 2020). Several studies presented below are in sports contexts and especially in team sports. Thus, in naturalistic approaches there is a need to connect decision-making and team cognition research in both athletes (Steiner et al., 2017; Bourbousson et al., 2019), referees (Boyer et al., 2020) and coaches (Richards et al., 2017). Three perspectives could be investigated. Firstly, Kermarrec and Bossard (2014) showed that decision-making in defensive phases in soccer need to be coordinated. Results support the fact that decision-making is connected to shared cues in the situation. Secondly, as this paper argued for the investigation of the development of IDM to understand how athletes become experts, there is a need to understand the development of collective intuition at team level (Kérivel et al., 2021). Future studies could use recent theoretical advances (Akinci and Sadler-Smith, 2019) to understand links between collective intuition and decision-making. Thirdly, this paper suggests that IDM could be influenced by emotions. From a team perspective, shared emotion could also influence team naturalistic decision-making and the interpersonal coordination between team members (Van Hoorebeke, 2006; Tamminen and Crocker, 2013). Future studies may examine the influence of emotions on sharing and decision-making processes in team sports, coaching teams, and referee teams.

## Conclusion

The use of the NDM approach, and more specifically of the RPD model is relatively recent in the field of sport. The growing number of empirical studies has confirmed its relevance for the understanding and enrichment of decision-making in sports settings. Previous results also invite researchers to question the model and its relations with six other determinants of expertise (analytical decisions, visual search strategies, creativity, emotions, development and team coordination), usually studied separately from IDM in both athletes, referees and coaches. In conclusion, the six avenues of research to revisit the RPD model, as suggested in this article, require innovative research methods to capture the complexity of IDM in naturalistic sports contexts.

## Author Contributions

All authors listed have made a substantial, direct, and intellectual contribution to the work and approved it for publication.

## Conflict of Interest

The authors declare that the research was conducted in the absence of any commercial or financial relationships that could be construed as a potential conflict of interest.

## Publisher's Note

All claims expressed in this article are solely those of the authors and do not necessarily represent those of their affiliated organizations, or those of the publisher, the editors and the reviewers. Any product that may be evaluated in this article, or claim that may be made by its manufacturer, is not guaranteed or endorsed by the publisher.
